# Distal Biceps Reconstruction With Achilles Tendon Allograft Using Transosseous Suture Fixation

**DOI:** 10.1016/j.eats.2025.103544

**Published:** 2025-04-04

**Authors:** Tyler R. Mange, Ryan S. Beyer, Misha H. Seifi, Tyler R. Johnston, Jesse D. Kaplan

**Affiliations:** From the Department of Orthopaedic Surgery, University of California Irvine, Orange, California, U.S.A.

## Abstract

Given the biceps brachii muscle’s importance for upper-extremity function, particularly forearm supination strength and elbow flexion, operative treatment of distal biceps tendon ruptures is typically strongly considered. When primary tendon repair is not possible, multiple surgical techniques exist for reconstruction of the distal biceps insertion. These techniques vary in terms of both fixation options and graft choices. In this article, we describe a technique for distal biceps reconstruction using Achilles tendon allograft, secured to the radius via bone tunnels, that requires no specialized equipment and restores the anatomic distal biceps insertion. This technique is therefore broadly applicable and results in a consistent, robust reconstruction to restore forearm supination strength throughout the range of motion.

Distal biceps brachii tendon ruptures typically occur in middle-aged men as a result of rapid, eccentric extension of the supinated elbow.^1^ From a biomechanical perspective, the distal biceps brachii tendon plays a crucial role in maintaining functionality of the upper extremity, specifically contributing to forearm supination and elbow flexion strength. Therefore, operative intervention for distal biceps ruptures is indicated in most cases to restore supination, as well as elbow flexion strength and endurance.^2^, ^3^, ^4^ Primary repair is the mainstay of operative management of distal biceps ruptures, with numerous techniques and fixation options described as means to restore the anatomic tendon insertion footprint, which has been shown to be important.^5^, ^6^, ^7^

In some cases, including more proximal tears and chronic injuries with significant proximal retraction, primary repair may not be possible. In these situations, reconstruction with allograft tissue is a viable method to bridge the gap from the residual tendon stump to the radial tuberosity.^1^^,^^8^ As in direct repair, multiple fixation devices and graft options have been suggested, each with proposed advantages.^8^, ^9^, ^10^ In this article, we describe a technique for distal biceps tendon reconstruction using Achilles tendon allograft secured via transosseous sutures (Video 1, Table 1). This technique requires no anchors or specialized equipment, restores the anatomic insertion of the distal biceps, and preserves the cam effect of the radial tuberosity, thus optimizing potential supination strength throughout the full range of forearm motion.^4^^,^^11^^,^^12^

**Table 1:** Pearls and Pitfalls of Surgical Technique

Pearls	Pitfalls
If the need for reconstruction is uncertain, begin with just the distal aspect of the incision.	Ensure that the lateral antebrachial cutaneous and posterior interosseous nerves are protected.
Approximate the insertions of the long and short heads of the biceps with each drill tunnel.	Avoid placing the drill tunnels too close to each other or too ulnar to minimize the risk of fracture.
Use an 18-gauge needle to pass the shuttle sutures on the dorsal aspect of the radius, and use a right-angle clamp to retrieve these sutures.	Confirm that the tourniquet is deflated and the elbow is at 60° of flexion prior to setting allograft tension.
Secure each of the allograft suture strands to the shuttle suture a few centimeters apart to allow for easier bone tunnel passage.	Check for gapping of the reconstruction with elbow range of motion prior to closure.

## Surgical Technique

### Preoperative Evaluation

Suspected distal biceps rupture is confirmed by history, physical examination, and imaging findings. Patients usually note a painful pop with an acute eccentric loading injury, but some may present months later with nonspecific elbow pain and weakness after an “arm strain” episode. Examination findings may include reverse Popeye deformity, positive hook test findings, and weakness with elbow flexion and forearm supination. Imaging assessment should include standard radiographs (often normal), as well as magnetic resonance imaging, to confirm the diagnosis, level of injury, and degree of retraction. When an injury is more chronic with retraction and/or minimal proximal tendon stump remains, the need for reconstruction is more likely. In our experience, injuries treated surgically more than 4 weeks after onset should be considered at high risk of requiring allograft reconstruction.

### Patient Positioning and Approach

**Fig 1:**
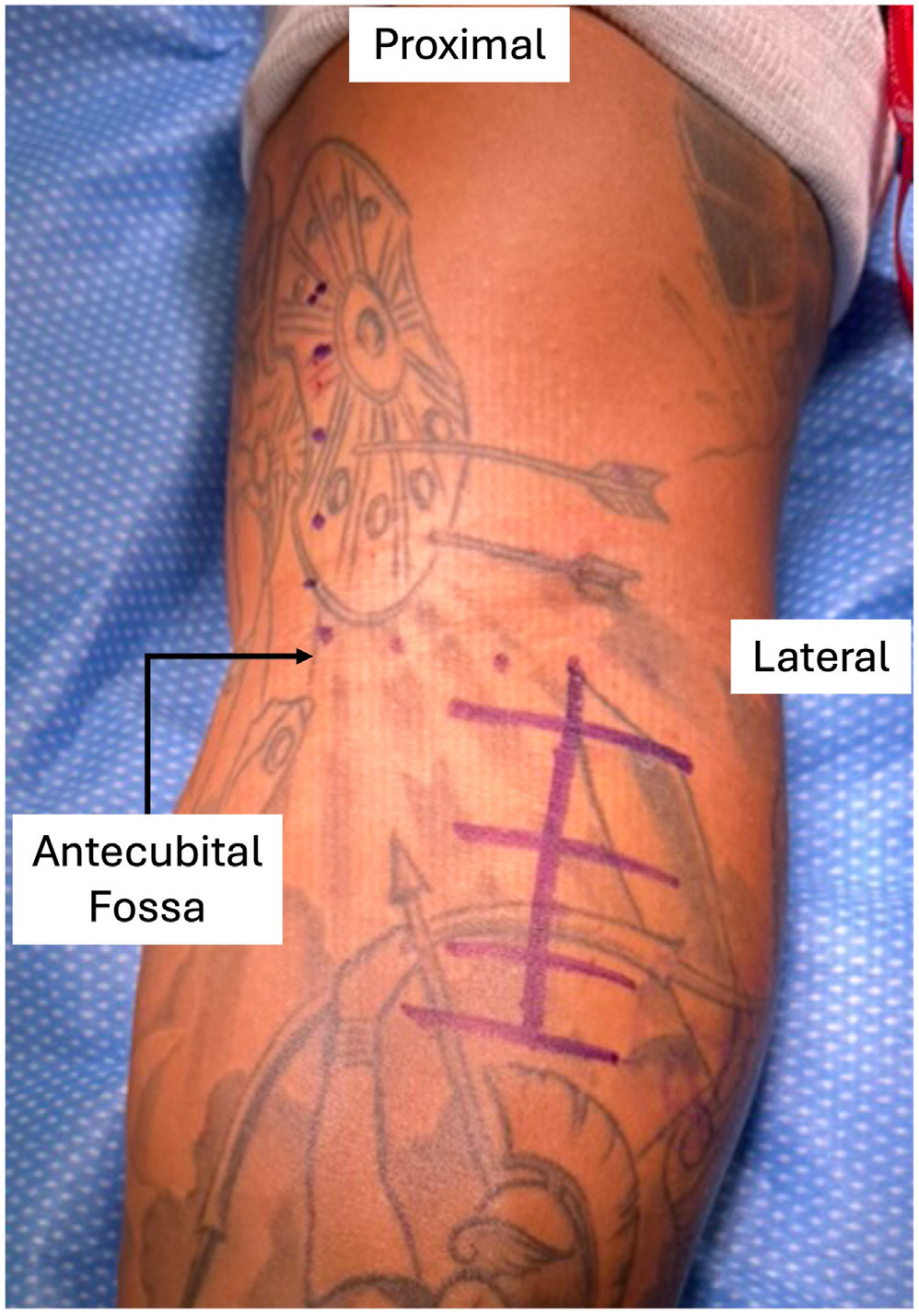
Marking of standard surgical incision (including purple dots and lines) for left-sided procedure with patient supine, arm on hand table, and pneumatic tourniquet on arm. The distal limb of the incision can be made first, prior to verifying that reconstruction (vs repair) is required.

The patient is positioned supine with the operative arm on a hand table. The upper extremity is prepared and draped, and a sterile tourniquet is then placed proximally on the arm. The tourniquet is inflated after the limb is exsanguinated. One gram of tranexamic acid is recommended prior to incision to help with hemostasis once the tourniquet is released. An S-shaped incision is marked, starting proximal and medial, extending transversely along the skin fold of the antecubital fossa, and continuing distally along the anterolateral forearm (Fig 1). The distal limb of the incision can be made first to explore the injury and degree of tendon retraction to confirm that reconstruction is necessary prior to extending the incision proximally. The dissection is performed along the interval between the pronator teres and brachioradialis, centered over the bicipital tuberosity, while the lateral antebrachial cutaneous nerve is identified and protected throughout the case. The forearm is maintained in supination to protect the posterior interosseous nerve. The biceps stump is identified and carefully cleared of soft-tissue adhesions proximally to maximize excursion. Any unhealthy scar tissue is then excised from the stump, and a Krackow traction suture is placed with No. 5 FiberWire (Arthrex, Naples, FL) or FiberLoop (Arthrex) for control and manipulation. At this point, if tendon excursion to the bicipital tuberosity is not possible, even with maximal elbow flexion, allograft reconstruction is indicated.

### Bone Tunnel Placement and Suture Passing

**Fig 2:**
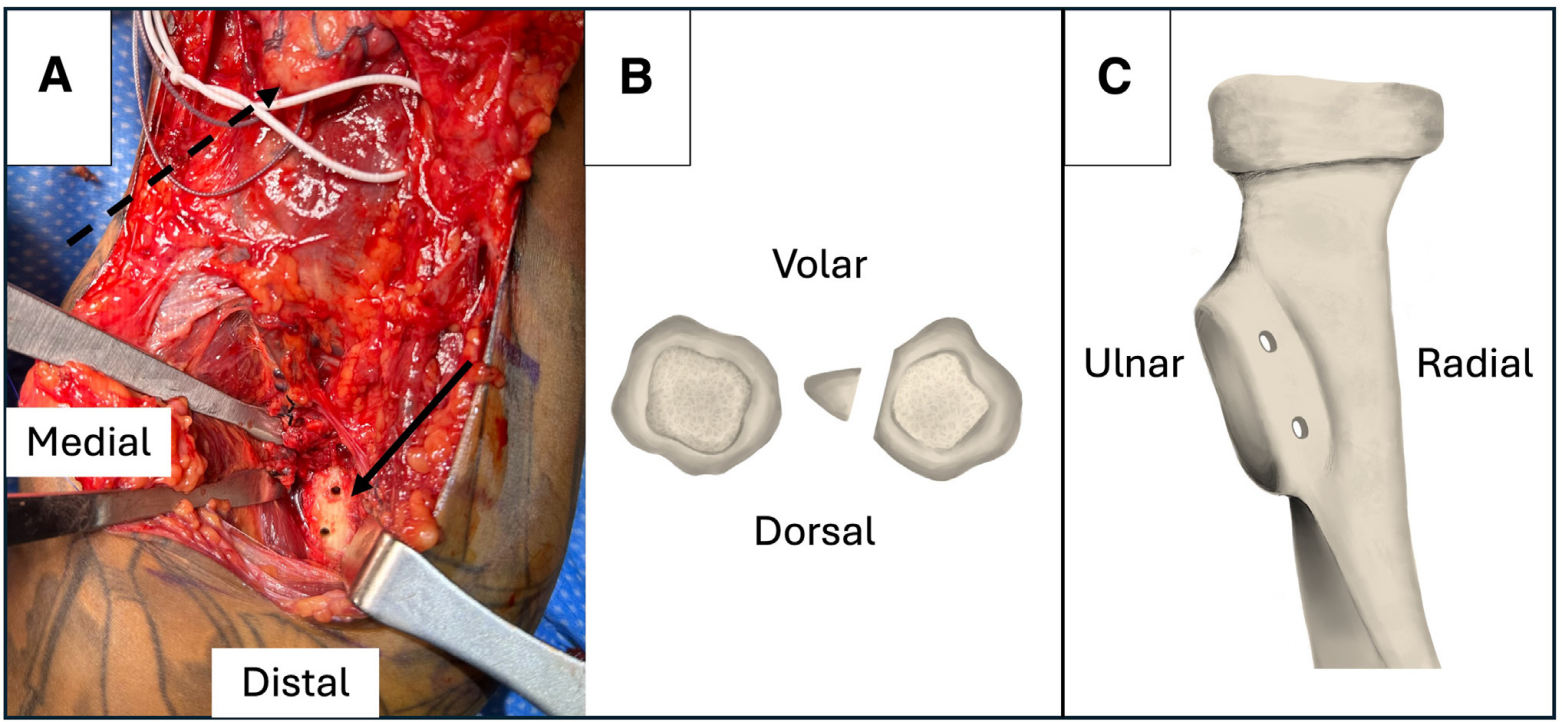
(A) Exposed radial tuberosity (solid arrow) with 2.5-mm drill tunnels at approximate insertion points of short and long heads of biceps. The biceps is retracted proximally in the wound (dashed arrow), and a vessel loop (white) is placed around the lateral antebrachial cutaneous nerve. (B, C) Orientation of radial tuberosity drill tunnels, viewed in axial plane and from volar to dorsal, for left-sided procedure.

Retractors are placed around the bicipital tuberosity for visualization, and the tuberosity is cleared and debrided using an elevator to expose healthy, bleeding bone. The footprints of both heads of the biceps should be identified to guide bone tunnel placement. Once adequately prepared, 2 tunnels are drilled in the tuberosity from anterior to posterior using a 2.5-mm drill in a slight ulnar-directed trajectory (Fig 2). Care is taken not to plunge the drill too deeply, and bone debris is removed from the wound. A No. 0 Prolene suture (Ethicon, Raritan, NJ) is passed through the back of an 18-gauge needle such that the suture exits at the tip of the needle. The needle is then placed from anterior to posterior through 1 bone tunnel. The suture is advanced slightly while a right-angle clamp, placed posteriorly from the medial aspect of the tuberosity, is used to grasp the tip of the needle on the far side of the tunnel. The needle is then slowly withdrawn while the clamp is tightened, grasping the transosseous suture, which is subsequently pulled from the wound. The suture ends are clamped together for later shuttling purposes. This process is repeated for the second tunnel. This insertion technique is adapted from the single-incision power-optimizing cost-effective primary repair technique.^11^

### Allograft Preparation

**Fig 3:**
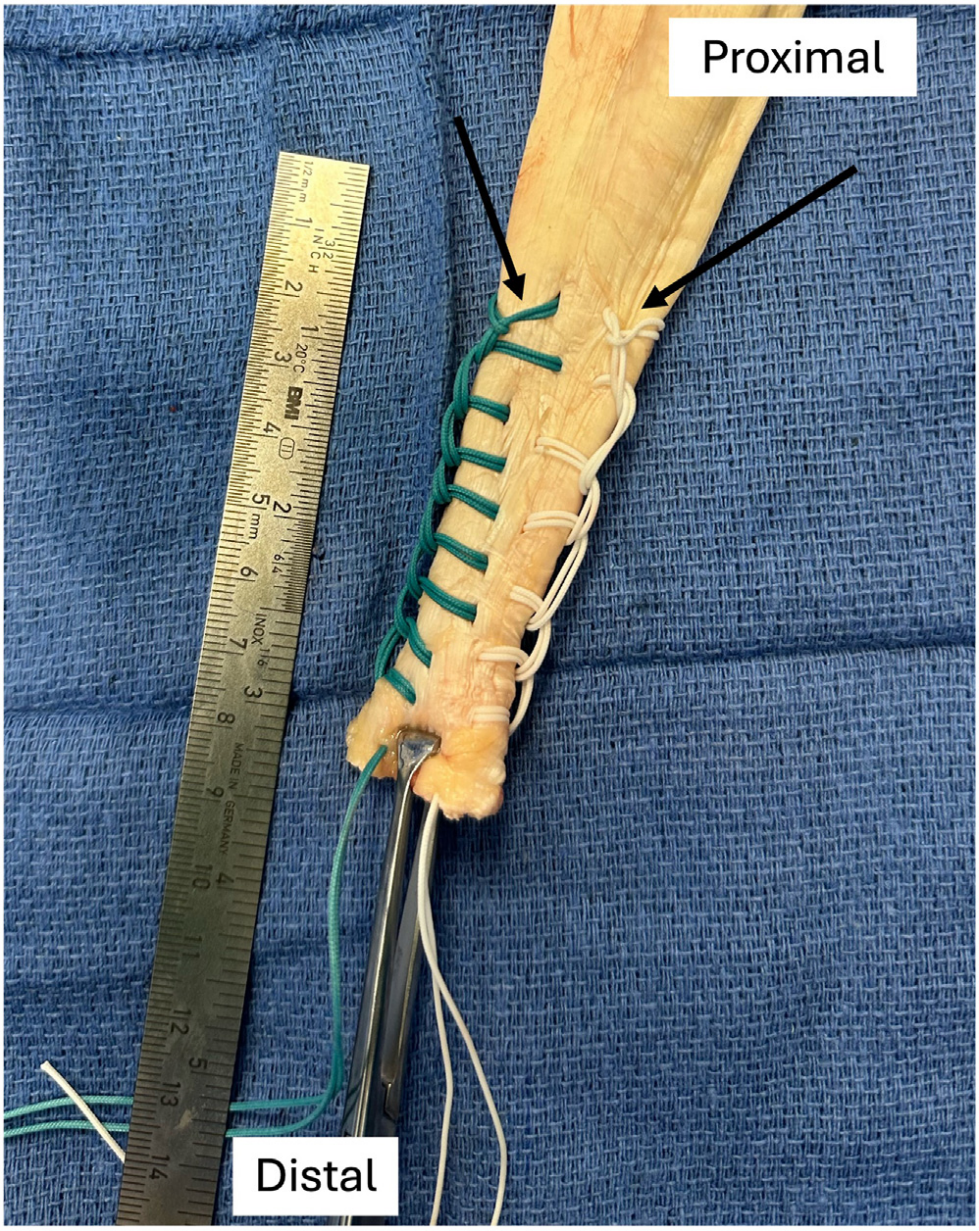
Preparation of distal aspect of allograft Achilles tendon using looped sutures (Nice Loops) via running, locking technique (arrows) on each side of tendon.

**Fig 4:**
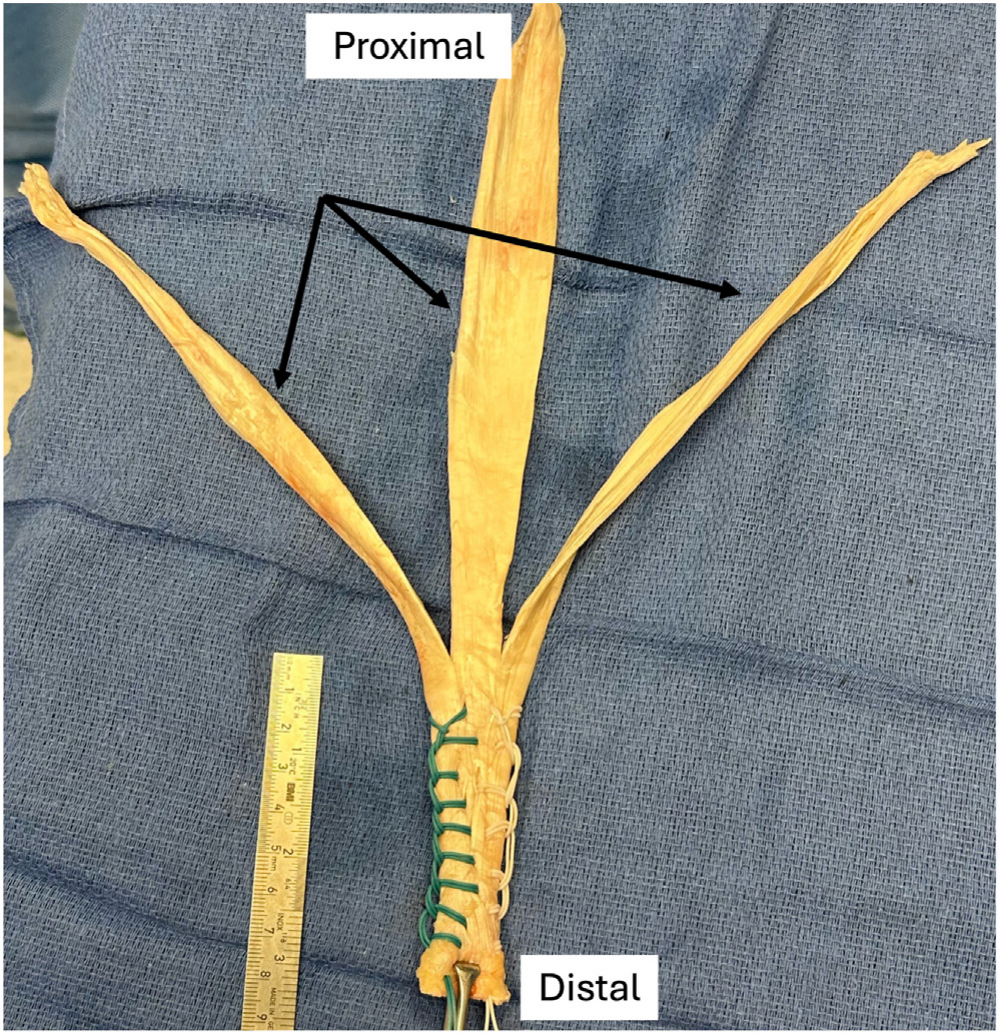
Preparation of proximal allograft Achilles tendon by division into 3 equal, longitudinal segments (arrows).

**Fig 5:**
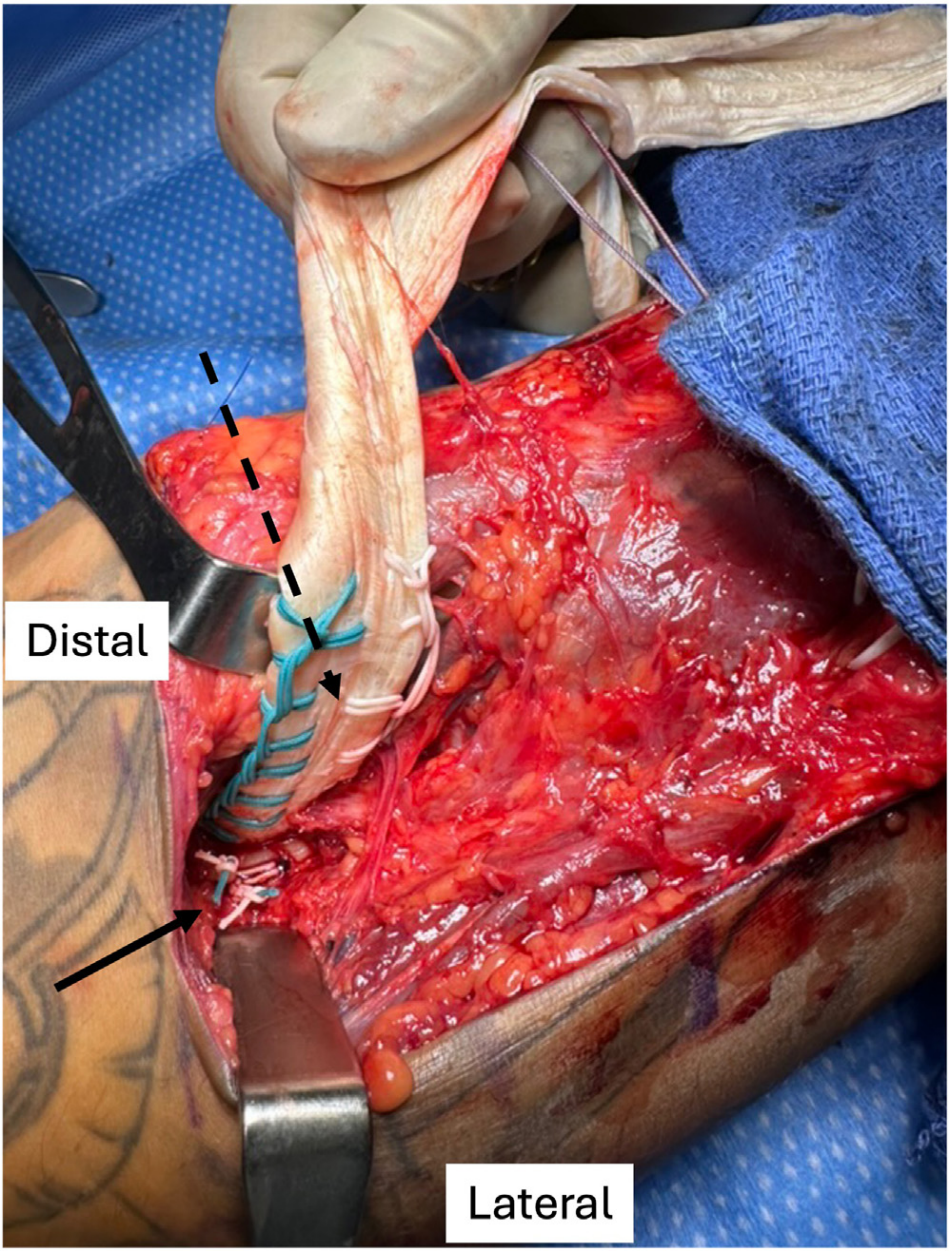
Distal allograft tendon (dashed arrow) secured to posterior aspect of radial tuberosity during left-sided procedure. One suture limb from each side of the allograft (i.e., 1 green and 1 white suture) is paired and tied over the anterior bone bridge while pulling tension with the other limbs (solid arrow). The second pair is then tied to complete the fixation of the allograft to the radius.

The distal end of an Achilles allograft is prepared using 2 No. 5 Nice Loops (Stryker, Kalamazoo, MI) of different colors in a running, locking fashion, leaving 2 suture tails on each side of the distal tendon (Fig 3) and then removing the needles. The proximal, fan-shaped expansion of the allograft tendon is split longitudinally into 3 portions (Fig 4). Once this is completed, 1 of the transosseous PDS shuttle sutures (Ethicon) is used to pass both suture limbs from 1 side of the allograft (i.e., both white sutures in Fig 3) through 1 bone tunnel in the radius. This is most easily accomplished by separately tying the suture limbs within the PDS shuttle suture rather than trying to pass both sutures together. The second shuttle suture is subsequently used to pass the remaining (other color) suture limbs through the other tunnel. The sutures are paired, with 1 suture from each of the proximal and distal tunnels (1 of each color), and 1 pair is used to hold tension while the other is tied tightly over the anterior bone bridge with alternating half-hitches and switching posts, securing the allograft tendon to the posterior aspect of the radial tuberosity (Fig 5).

**Fig 6:**
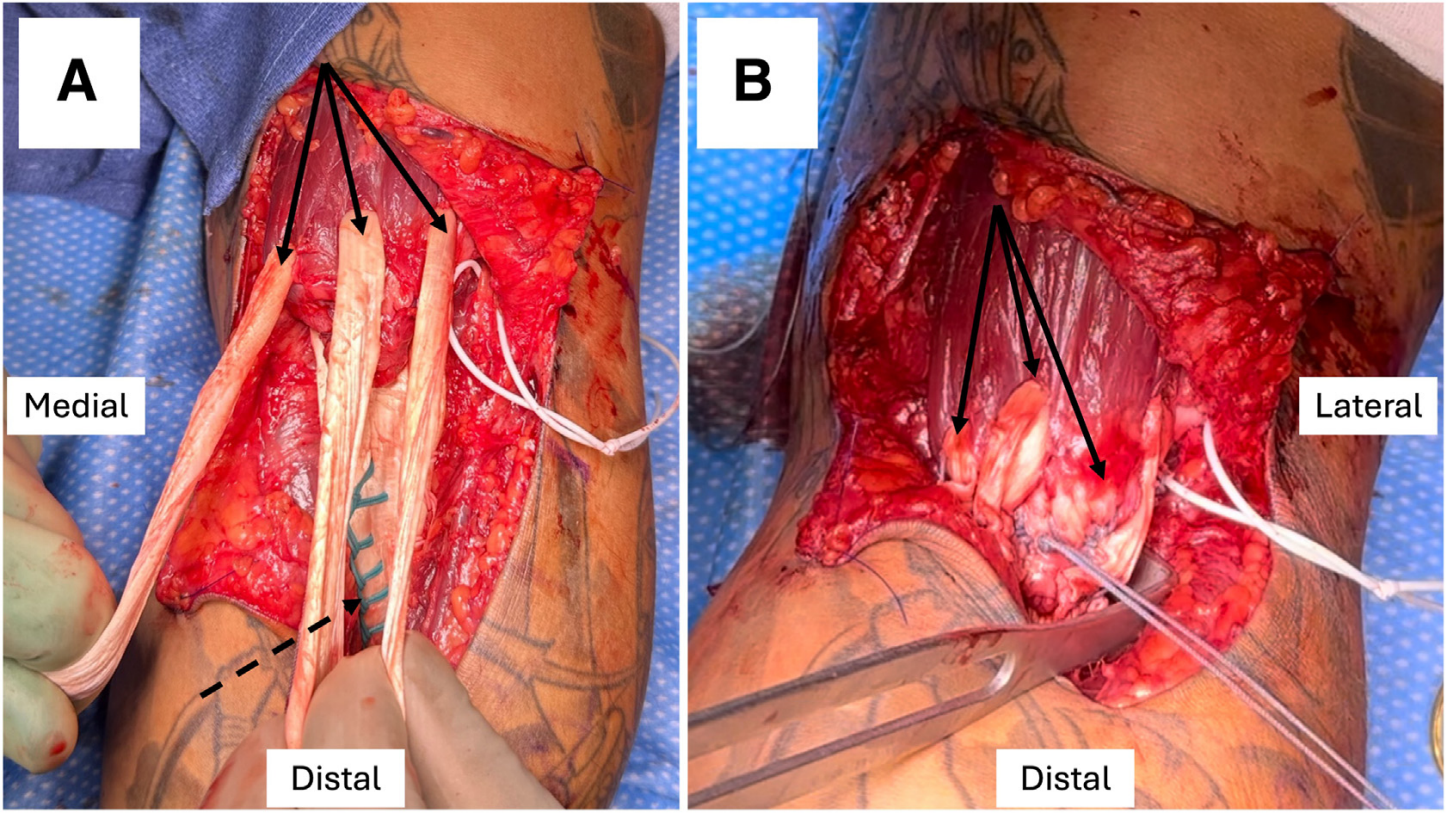
(A) Proximal segments of allograft tendon (solid arrows) looped through biceps just proximal to myotendinous junction in 3 areas (medial, central, and lateral) for left-sided procedure. The medial allograft segment should correspond to the anterior aspect (dashed arrow) of the distal allograft tendon, which is secured at the more distal drill tunnel in the radial tuberosity. (B) Proximal allograft segments (solid arrows) subsequently sutured to distal biceps stump and distal allograft tendon.

Next, the elbow is placed in 60° of flexion. The 3 segments of the proximal aspect of the allograft are passed directly through the biceps muscle proximal to the musculotendinous junction from posterior to anterior in 3 different places: medial, central, and lateral (Fig 6A). This is facilitated by passing a tonsil clamp through the muscle from anterior to posterior to grasp the tendon ends. The reconstruction is oriented for anatomic reconstruction such that the more medial short head of the biceps will insert more distally on the bicipital tuberosity. These segments are then looped back onto themselves distally, pulled taut, and sutured to themselves using No. 2 FiberWire suture (Arthrex) with figure-of-8 stitches (Fig 6B). The tourniquet is deflated just prior to tensioning. Once secured in appropriate tension, 2 additional suture fixation points are placed in each allograft loop, and the No. 5 FiberWire traction suture is used to further reinforce the reconstruction using a free needle (Fig 6). At this point, the reconstruction is evaluated and confirmed to be moving as a unit with gentle elbow extension, verifying that no gapping is appreciable at either the tendon insertion or the muscle-allograft interface.

### Closure and Postoperative Care

The wound is irrigated, and hemostasis achieved with bipolar electrocautery. One gram of vancomycin powder is placed into the wound. The wound is closed in a layered fashion with absorbable sutures. The patient is placed in a posterior long arm splint in 90° of elbow flexion and supination. After 2 weeks, the patient is transitioned to a long arm cast (or a locked, hinged elbow brace) at 90° for an additional month before beginning a graduated range-of-motion program and supervised physical therapy at 6 weeks. Full, progressive range of motion is allowed initially, along with active-assisted elbow flexion. Isometric biceps exercises begin at 9 weeks with a 5-lb weight limit until 12 weeks. After 12 weeks, no restrictions remain, and the patient can begin progressive strengthening.

## Discussion

In certain cases, including chronic or proximal tears adjacent to the myotendinous junction, direct repair of a distal biceps rupture is not feasible, and allograft reconstruction is required. In this article, we describe a technique for allograft reconstruction using Achilles tendon and transosseous tunnels that requires no anchors or specialized equipment. In our experience, this technique creates reproducible, robust fixation of the allograft to both the radial tuberosity and the residual biceps proximally, with anatomic reconstruction of the biceps musculotendinous junction and tendon insertion. The two 2.5-mm bone tunnels in the bicipital tuberosity are small and therefore minimize fracture risk. Additionally, there is no disruption of the normal radial tuberosity prominence, which has been shown to help maintain the supination moment arm.^6^ By dependably re-creating the biceps insertion on the posterior aspect of the radial tuberosity, this technique offers a means to optimize supination strength throughout the full range of forearm rotation.^4^^,^^11^^,^^12^ In summary, the described methodology for distal biceps allograft reconstruction requires no specialized equipment (making it practical and widely accessible) and creates a reproducible and robust reconstruction to optimize postoperative biceps muscle function.

## Disclosures

All authors (T.R.M., R.S.B., M.H.S., T.R.J., J.D.K.) declare that they have no known competing financial interests or personal relationships that could have appeared to influence the work reported in this paper.

## Supplementary Data

Video 1 After positioning of the patient supine with the operative left arm on a hand table, preparation and draping, and insufflation of a sterile arm tourniquet, an anterior approach to the antecubital fossa is performed using an S-shaped incision. The lateral antebrachial cutaneous nerve is protected, and the distal biceps brachii tendon is exposed and mobilized. The radial tuberosity is exposed via the interval between the pronator teres and brachialis and cleared of soft tissue. If the biceps tendon cannot be mobilized to the radial tuberosity, even with elbow flexion, reconstruction is indicated. Two 2.5-mm bone tunnels are drilled in the radial tuberosity, one at the normal insertion of the short head of the biceps and the other at the long head insertion. The distal aspect of the Achilles tendon allograft is prepared using 2 running, locking sutures that are then passed through the bone tunnels from posterior to anterior and tied over the anterior bone between the 2 tunnels. The proximal aspect of the allograft is split into 3 equal, longitudinal segments and looped through the biceps muscle from posterior to anterior in 3 distinct places (medial, central, and lateral). The loops are then brought back distally and secured to the distal portion of the allograft with nonabsorbable sutures, ensuring appropriate tensioning of the reconstruction at this time. The reconstruction is reinforced with additional suture. The elbow is then moved through its range of motion to ensure that there is no gapping at both the bone-allograft and allograft-muscle interfaces. Finally, a layered closure is performed, and the patient is placed into a long arm splint.
